# Supplementary material for: Methanol-essential growth of Escherichia coli
Source: Nat Commun. 2018 Apr 17;9:1508. doi: 10.1038/s41467-018-03937-y (PMC5904121; doi:10.1038/s41467-018-03937-y)
Supplement: Supplementary file 1 — Supplementary Information [file 41467_2018_3937_MOESM1_ESM.pdf]

# Supplementary Information

# Methanol-essential growth of *Escherichia coli*

**Fabian Meyer, Philipp Keller, Johannes Hartl, Olivier G. Gröninger, Patrick Kiefer, Julia A. Vorholt**

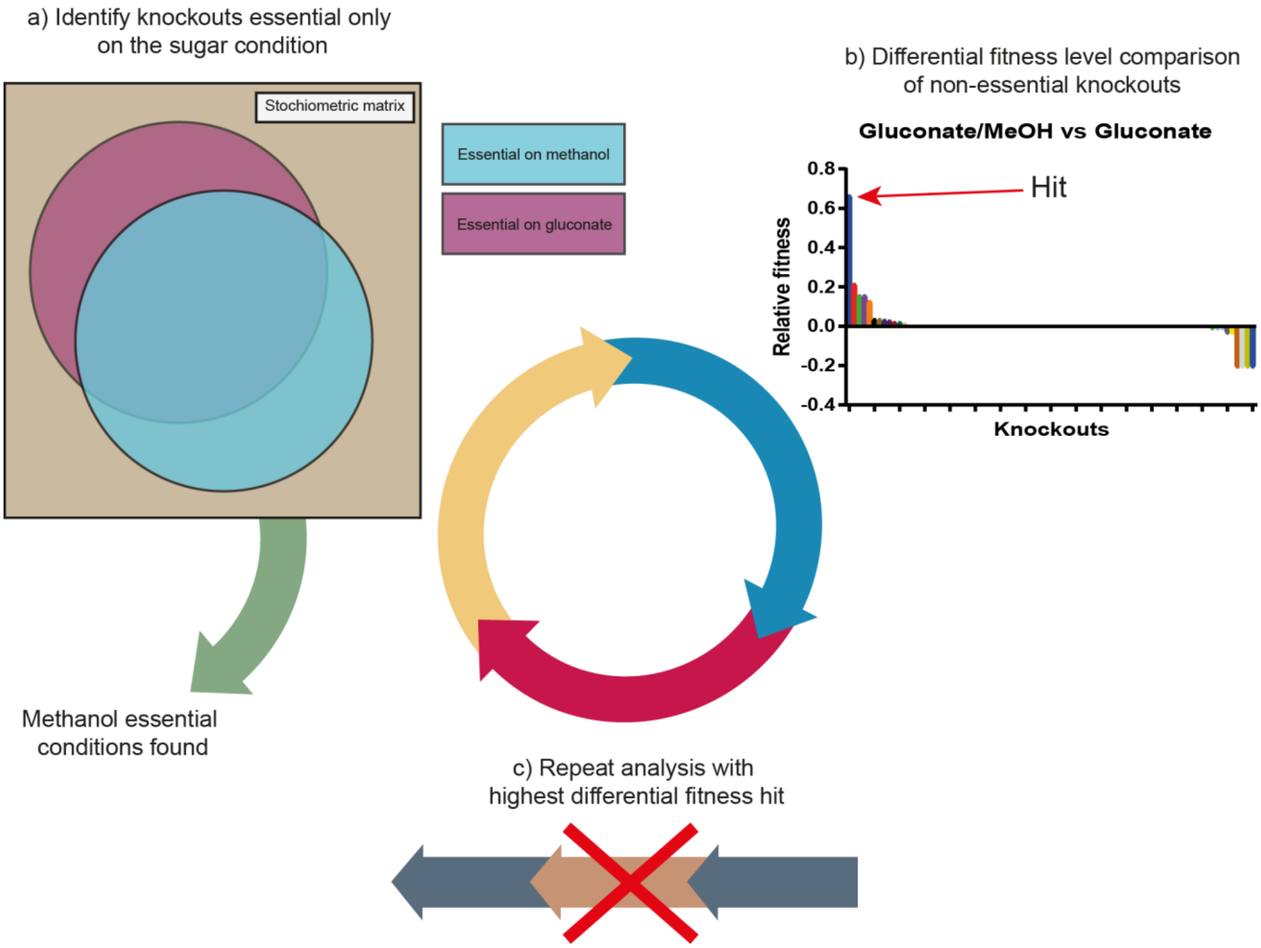


**Supplementary Figure 1**. Iterative workflow to identify methanol-essential knockouts, gluconate is chosen as case study. a) As a first step, knockouts that are essential only on the sugar condition are identified. b) In case no knockout can be found, differential fitness level comparison of non-essential knockouts is performed comparing predicted growth rates of gluconate vs gluconate/methanol condition. c) Analysis is repeated with highest differential fitness hits until methanol-essential genotype is identified.

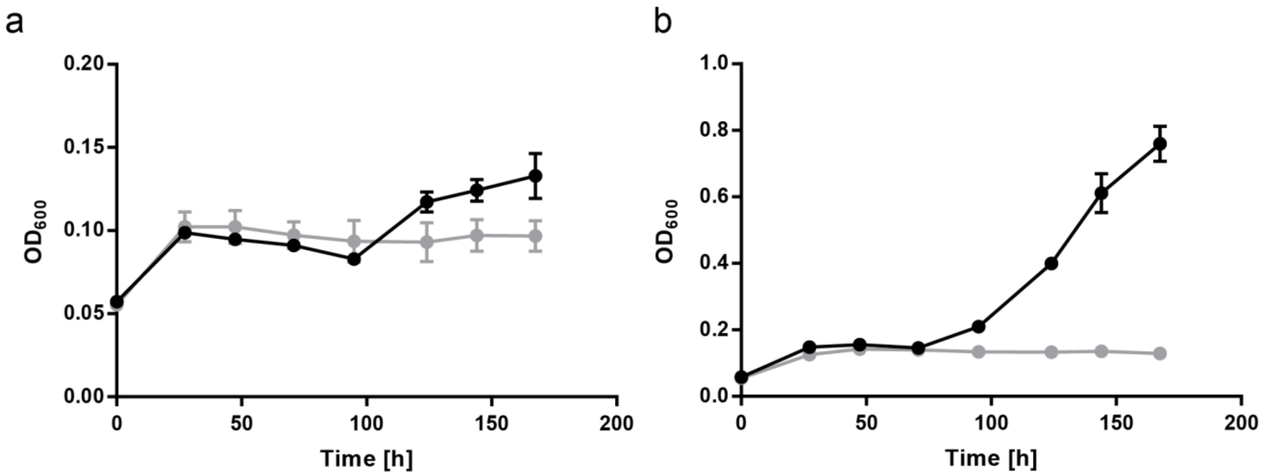


**Supplementary Figure 2** Initial growth experiment of methanol-essential strain version 1 (n=3). a) Initial growth experiment on 5 mM gluconate (0.1 g $L^{-1}$ yeast extract) with (black) and without (grey) 500 mM methanol. b) Initial growth experiment on 5 mM gluconate and 20 mM pyruvate (0.1 g $L^{-1}$ yeast extract) with (black) and without (grey) 500 mM methanol. Data presented as mean ± standard deviation.

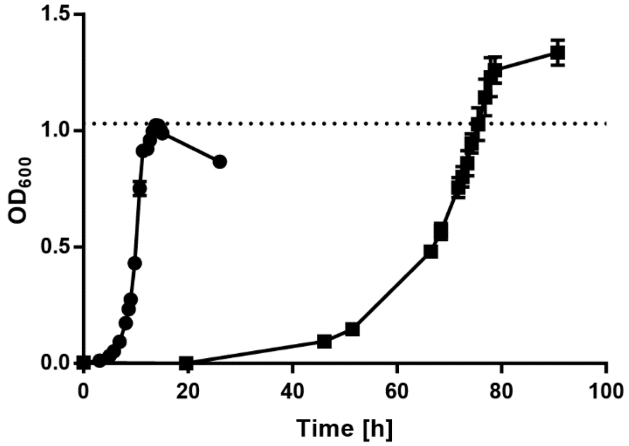


**Supplementary Figure 3** Growth comparison between MeSV2.2 and wild type *E. coli* with empty plasmids. Growth curves of MeSV2.2 (squares) and wild type *E. coli* strain containing empty plasmids (circles) under the same growth conditions; 5 mM gluconate with 500 mM methanol and without yeast extract. Data presented as mean ± standard deviation (n=3).

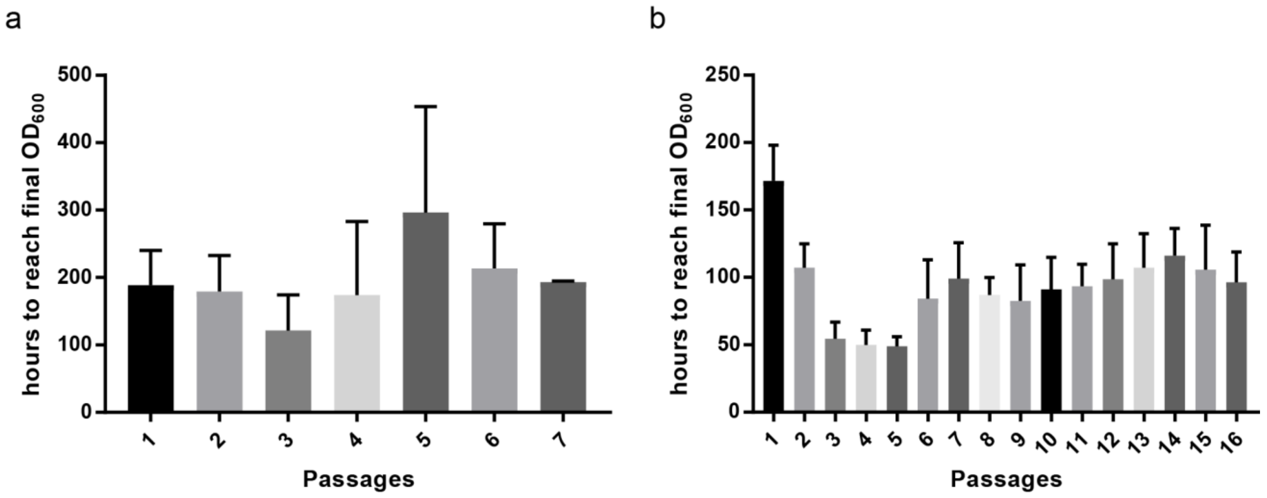


**Supplementary Figure 4** Average time in hours to reach final $OD_{600}$ per passage in case of evolution experiments of MeSV1.1 and MeSV2.1. Data presented as mean ± standard deviation. a) In case of MeSV1.1 (n=5) and b) in case of MeSV2.1 (n=6).

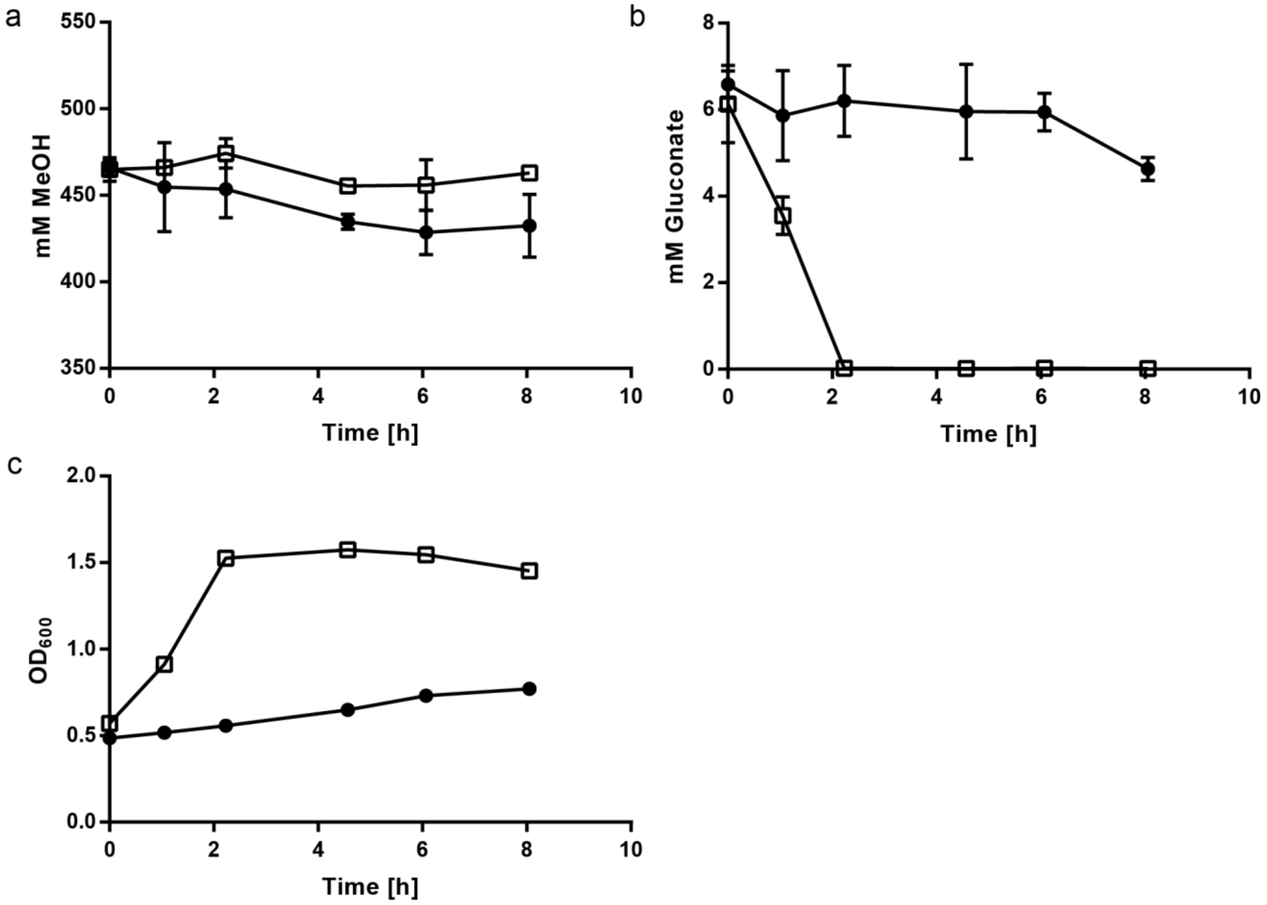


**Supplementary Figure 5** Methanol and gluconate consumption of MeSV2.2 (black circles) and wild type *E. coli* containing empty vector control (open squares); n=3. Data presented as mean ± standard deviation. a) Methanol consumption b) gluconate consumption c) growth during consumption.

**Supplementary Table 1:** Oligonucleotides used in this study.

| Oligonucleotides: | Sequence: |
|---|---|
| 1_Fwd_Mdh2_*Bacillus_methanolicus*_PB1 | GGGGAATTCAGGAGATATACATATGACAAACACTC |
| 2_Rev_Mdh2_ *Bacillus_methanolicus*_PB1 | GGGCTGCAGTTACTCGAGCATAGCATTTTTAATAAT |
| 3_Fwd_Hps_Phi_*Methylobacillus_flagellatus* | GGGGAATTCAGGAGAAAGTATCGTGGCATTGAC |
| 4_Rev_Hps_Phi_*Methylobacillus_flagellatus* | GGGAAGCTTTTATTCAAGATTGGCGTGAA |
| 5_rpiA_seq_fw | CGTTGCCCAGCGGCAGTTTATTTTC |
| 6_rpiA_seq_rv | CTACCTGCAAGTTGAGGCGGATAG |
| 7_rpiB_seq_fw | ATCCTCCGGCACCGTACAAATC |
| 8_rpiB_seq_fw | GCAGGTAATGGTGATGGATTGC |
| 9_edd_seq_fw | GCTCCGGTTACAGGCGTTTCAG |
| 10_edd_seq_rv | CACGATAACCGGTACAACCGGG |
| 11_maldh_seq_fw | GGCAGCGGAGCAACATATC |
| 12_maldh_seq_rv | TCTGTGCTCCGGTTTTTTATTATCC |
| 13_pSEVA_seq_fw | CATCCGGCTCGTATAATGTG |
| 14_pSEVA_seq_rv | GAGTTCTGAGGTCATTACTG |

**Supplementary Table 2:** Strains used in this study.

| Strain | Genotype | Description | Reference |
|---|---|---|---|
| **Wild type empty plasmids** | BW25113[3] pSEVA424 pSEVA131 | Wild type *E. coli* strain used as control | This study |
| **Wildtype pSEVA424 *mdh2* PB1 pSEVA131 *hps phi* M.f.** | BW25113 pSEVA424 *mdh2* PB1 pSEVA131 *hps phi* M.f. | Wild type *E. coli* strain harboring plasmids encoding *mdh2* PB1 and *hpsphi* M.f. used as comparison | This study |
| **MeSV1 (Methanol-essential strain)** | BW25113 Δ*rpiA*Δ*rpiB*Δ*edd* pSEVA424 *mdh2* PB1 pSEVA131 *hps phi* M.f. | Methanol dependent strain on gluconate. Parental strain of MeSV1.1 | This study |
| **MeSV2 (Methanol-essential strain version 2)** | BW25113 Δ*rpiA*Δ*rpiB*Δ*edd*Δ*maldh* pSEVA424 *mdh2* PB1 pSEVA131 *hps phi* M.f. | Methanol dependent strain on gluconate with reduced TCA cycle activity. Parental strain of MeSV2.1 and MeSV2.2 | This study |
| | | | |

**Supplementary Table 3:** Plasmids used in this study.

| Plasmid | Encoded gene | Description | Reference |
|---|---|---|---|
| **pSEVA424** | | Low-copy-number, lacIq/Ptrc promotor, RK2 ori, $Sm^R$. | (Silva-Rocha et al., 2013)[1] |
| **pSEVA131** | | Medium-copy-number, lacIq/Ptrc promotor, pBBR1 ori, $Amp^R$. Original pSEVA131 plasmid does not contain a promotor. | (Silva-Rocha et al., 2013)[1] |
| **pSEVA424 *mdh2* PB1** | *mdh2* gene (PB1_12584) of *B. methanolicus* PB1 | *mdh2* gene was amplified from pET21a mdh2 PB1 using primers 1 and 2 and ligated into the *EcoRI*/*PstI* site of pSEVA424 | (Müller et al., 2015)[2] |
| **pSEVA131 *hps phi* M.f.** | *hps* and *phi* genes (Mfla_1654 and Mfla_1653) of *M. flagellatus* KT (DSM 6875) | *hps phi* operon was amplified from genomic DNA using primers 3 and 4 and ligated into the *EcoRI*/*HindIII* site of pSEVA131 | This study |

**Supplementary References**


1. Martínez-Garćía, E., Aparicio, T., Goñi-Moreno, A., Fraile, S. & De Lorenzo, V. SEVA 2.0: An update of the Standard European Vector Architecture for de-/re-construction of bacterial functionalities. *Nucleic Acids Res.* **43,** D1183–D1189 (2015).

2. Müller, J. E. N. *et al.* Engineering *Escherichia coli* for methanol conversion. *Metab. Eng.* **28,** 190–201 (2015).

3. Baba, T. *et al.* Construction of *Escherichia coli* K-12 in-frame, single-gene knockout mutants: the Keio collection. *Mol. Syst. Biol.* **2,** 2006.0008 (2006)
