# Supplementary material for: Methanol-essential growth of Escherichia coli
Source: Nat Commun. 2018 Apr 17;9:1508. doi: 10.1038/s41467-018-03937-y (PMC5904121; doi:10.1038/s41467-018-03937-y)
Supplement: Supplementary file 2 — Description of Additional Supplementary Files [file 41467_2018_3937_MOESM2_ESM.pdf]

Descriptions of Additional Supplementary Files

File Name: Supplementary Data 1
Description: Methanol-essential genotypes - iAF1260

File Name: Supplementary Data 2
Description: Methanol-essential genotypes - iML1515

File Name: Supplementary Data 3
Description: Identified mutations in the different evolution experiments
